# Nursing personnel management during COVID-19 pandemic: An exemption trend in view of health reasons

**DOI:** 10.3389/fpubh.2022.961308

**Published:** 2022-10-28

**Authors:** Sruti Sharma, Navneet Dhaliwal, Sanjay Bhadada, Ashok Kumar, Sumit Kumar Sangat, Navin Pandey

**Affiliations:** ^1^Department of Hospital Administration, Post Graduate Institute of Medical Education and Research, Chandigarh, India; ^2^Department of Endocrinology, Post Graduate Institute of Medical Education and Research, Chandigarh, India

**Keywords:** COVID-19, nursing personnel management, medical conditions and vulnerable staff, clear guidelines, effective engagement

## Abstract

**Background:**

In the COVID-19 pandemic, the healthcare system faced unprecedented challenges with increased number of patients and limited resources. Managing nursing resource was a major challenge for hospital administration. They had to be on the frontline, but their safety was of paramount importance.

**Aim:**

This study aims to analyze the measures taken for the management and effective engagement of nursing personnel for deployment in the COVID area of the hospital and the exemption trend based on their health status.

**Methodology:**

A retrospective cross-sectional descriptive study was carried out to analyze the requests of nursing staff received for exemption of duty in COVID patient care areas. These requests were categorized and examined by the medical board constituted for this purpose. Microsoft Excel was used to interpret the results.

**Results:**

The study evaluated the health reasons of nursing officers on the basis of which exemption was given for deployment of nursing officers in COVID areas. They were mostly medical reasons (91.1%) and few personal reasons (8.77%). The majority suffered from diseases affecting two or more than two specialties. Out of 376 applications, 223 were exempted, 81 were not exempted, 13 were given short-term exemption, and 26 were shifted to administrative assignments. Thirty-three staff members were referred to an appropriate forum.

## Introduction

The COVID-19 pandemic took over the world like an unrelenting storm testing the resilience of healthcare systems everywhere. Healthcare systems were faced with unexpected influx of patients requiring treatment within limited resources. Healthcare providers at the front line faced numerous challenges. Different guidelines were laid down to equip COVID facilities with staff, essential equipment, and medicines. The nursing staff and other healthcare providers were under extraordinary pressure and played a pivotal role in providing patient care services 24 × 7 during the pandemic. At the same time, management of the nursing manpower emerged as one of the major challenges the world over. Various International and national guidelines were laid down to address the huge demand of nursing staff trained in managing communicable diseases and infection prevention.

The situation was no different in our 2,210 bedded tertiary care institute and research center. Although there was an unprecedented demand for nursing staff and every possible resource had to be utilized, it was equally important that the health and wellbeing of committed care providers were adequately safe guarded. “No country, hospital, or clinic can keep its patients safe unless it keeps its health workers safe” was a statement issued by the WHO in a news release. In our institute, this was done by providing PPE, provision of testing for COVID-19, training for infection prevention and control, and psychological care by counseling to mention a few. A lot more measures were suggested to protect the old and vulnerable members in our communities, but apparently, similar emphasis was not given in the case of healthcare workers including nurses. There is not much literature available on protocols in place for vulnerable healthcare workers and their deployment in COVID patient care areas. In the absence of any clarity, hospitals and organizations devised their own protocols to safeguard vulnerable staff (1). In our country, a national level advisory was issued by the Ministry of Health and Family Welfare, Government of India for pregnant/lactating mothers and immunocompromised healthcare workers to inform their medical condition to hospital authorities for posting only in non-COVID areas (2, 3). This was a move meant to minimize risk to staff while ensuring effective management of patient care services and was followed in our institute.

This study aims to analyze the measures taken for the management and effective engagement of nursing personnel for deployment in the COVID area of the hospital and the exemption trend based on their health status.

## Materials and methods

A retrospective cross-sectional descriptive study was carried out to analyze the requests received for exemption of duty. The guidelines issued by the Ministry of Health and Family Welfare were circulated to concerned officials for dissemination. Applications/requests received from the nursing staff for exemption of duty in COVID areas were examined. A medical board was constituted to examine the requests seeking exemption on medical grounds based on the issued guidelines. The requests were categorized. The proceedings of the medical board and all related office orders were studied. Requests that did not fall under the purview of the board were referred to an appropriate forum. Microsoft Excel was used to interpret the results.

### Ethical clearance

The institute ethical committee (intramural) approved the study with protocol in their meeting held on 22 March.2022, with reference no: IEC-INT/2022/study-29.

## Results

The institute caters to more than 100,000 IPD patients, and as expected of a tertiary center, patient care services to both COVID and non-COVID cases (new and follow up) have to be provided. During the pandemic, the allocation of nursing staff in COVID patient care areas was done according to nursing norms, area requirements, government guidelines, and in consultation with nursing manpower management committee. The number of duty shifts of nursing staff was increased from 3 to 4 because of difficulties associated with wearing of PPE. The number of off-duties was increased to enable staff to handle stress and burn out along with rotational deployment in COVID care areas. The physical OPD was scaled down drastically during pandemic peak and elective surgeries were stopped to enable the diversion of staff for COVID care.

A total of 376 requests were received from the nursing cadre staff for exemption from COVID area duties in a period of 6 months, i.e., 1 August 2020 to 31 January 2021. The reasons for these requests were examined by the medical board which consisted of specialists from Department of Medicine, Endocrinology, Orthopedics, Urology, Psychiatry and Hospital Administration along with the provision of co-opting any other expert as and when required. Out of the 376 nursing officers, 323 were women and 53 were men.

**Figure 1:**
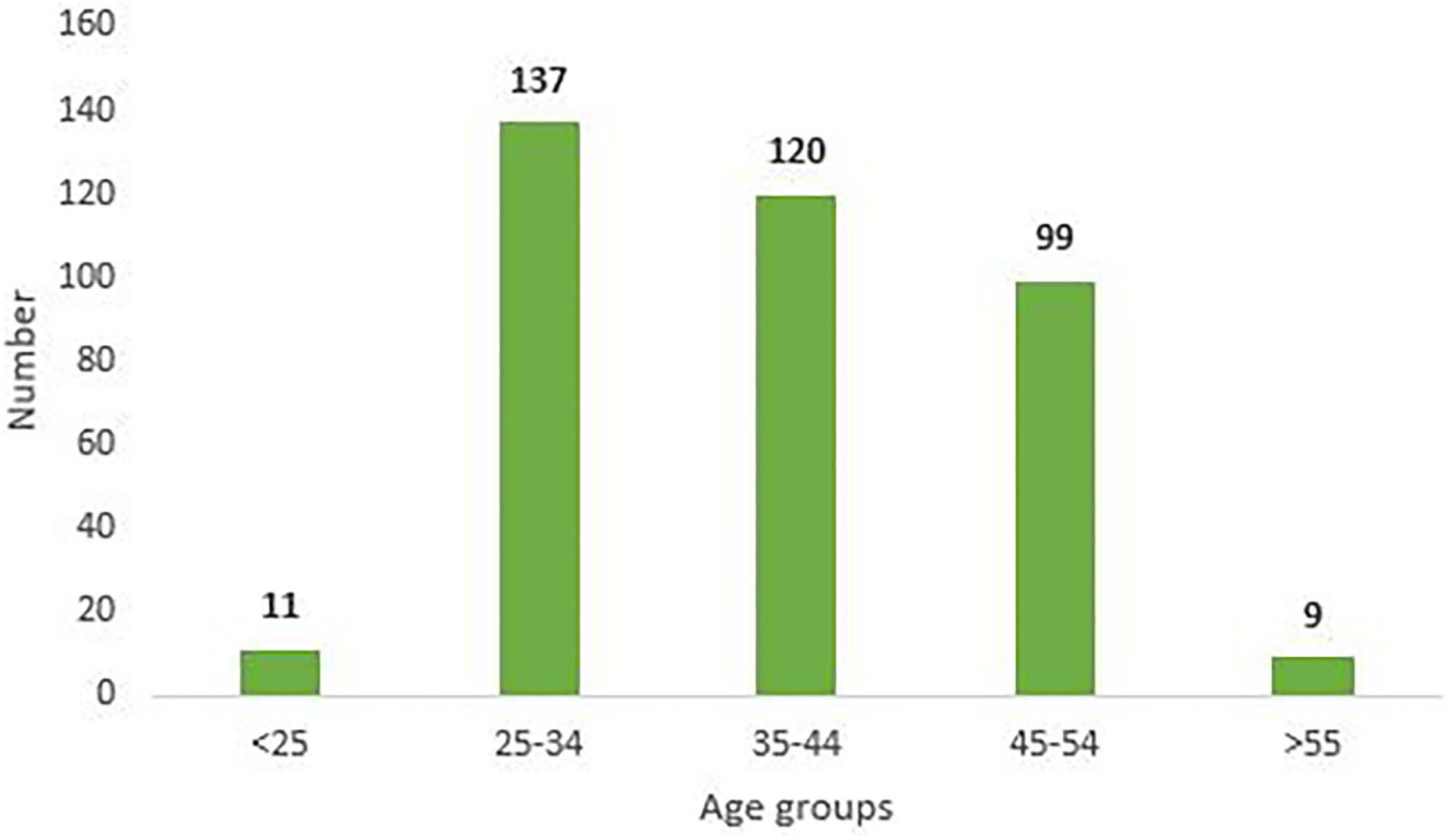
Age Wise distribution of requests for duty exemption in COVID care areas.

**Figure 2:**
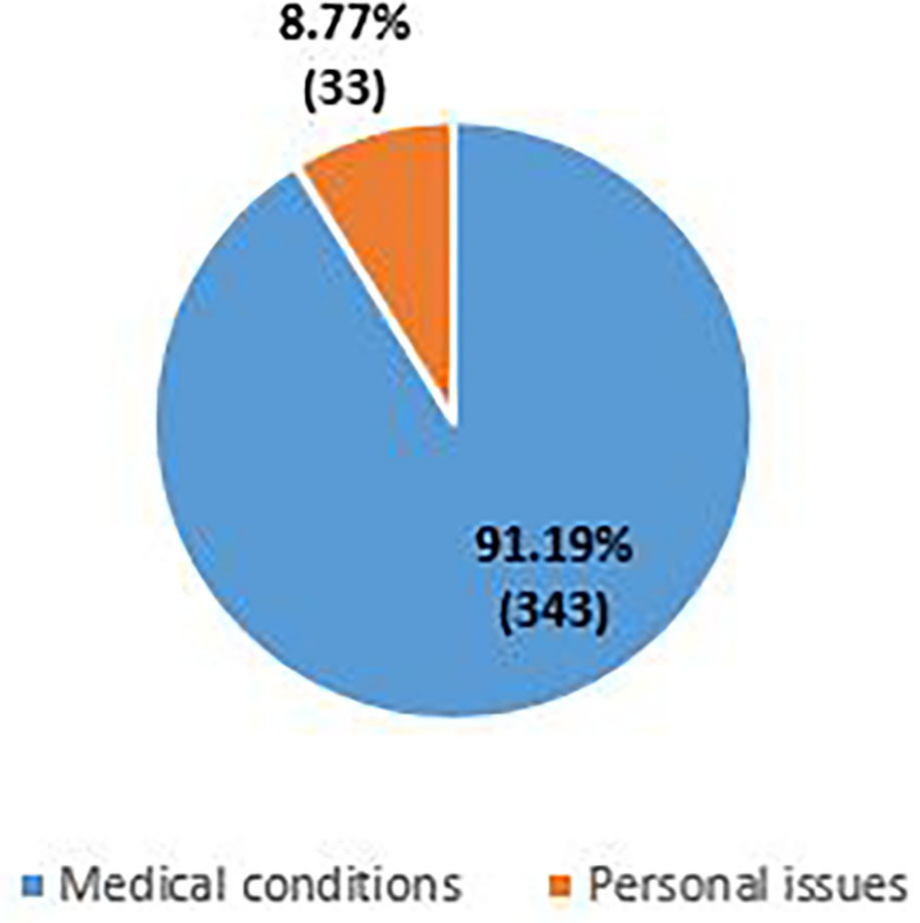
Categorization of requests.

**Figure 3:**
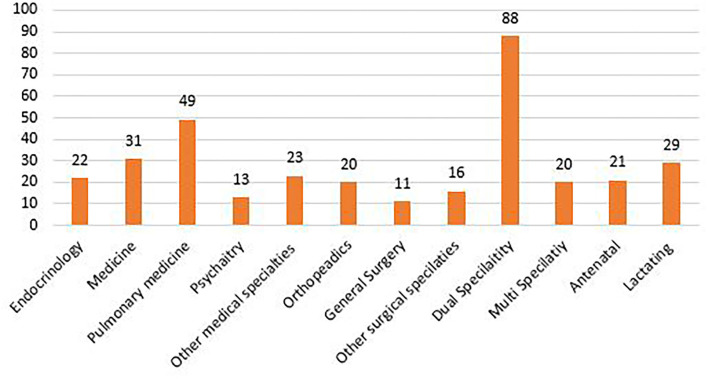
Categorization of exemption requests based on medical/surgical specialities.

The age wise grouping in Figure 1, shows that maximum number of Nursing officers who applied for exemption were in the age range of 25–34 years (36.4%), followed by 35–44 years (31.9%), 45–54 years (26.3%) and least in the age group <25 (2.9 %) and >55 (2.3%). The requests were examined and divided into two categories, namely, medical conditions (91.9%) and personal issues (8.77%), as seen in Figure 2. Most of the applicants were found to have diseases involving dual speciality (*N* = 88), followed by diseases involving pulmonary function (*N* = 49), internal medicine (*N* = 31), orthopedics, etc. Antenatal (21) and lactating (29) staff members were also included under medical conditions (Figure 3).

**Figure 4:**
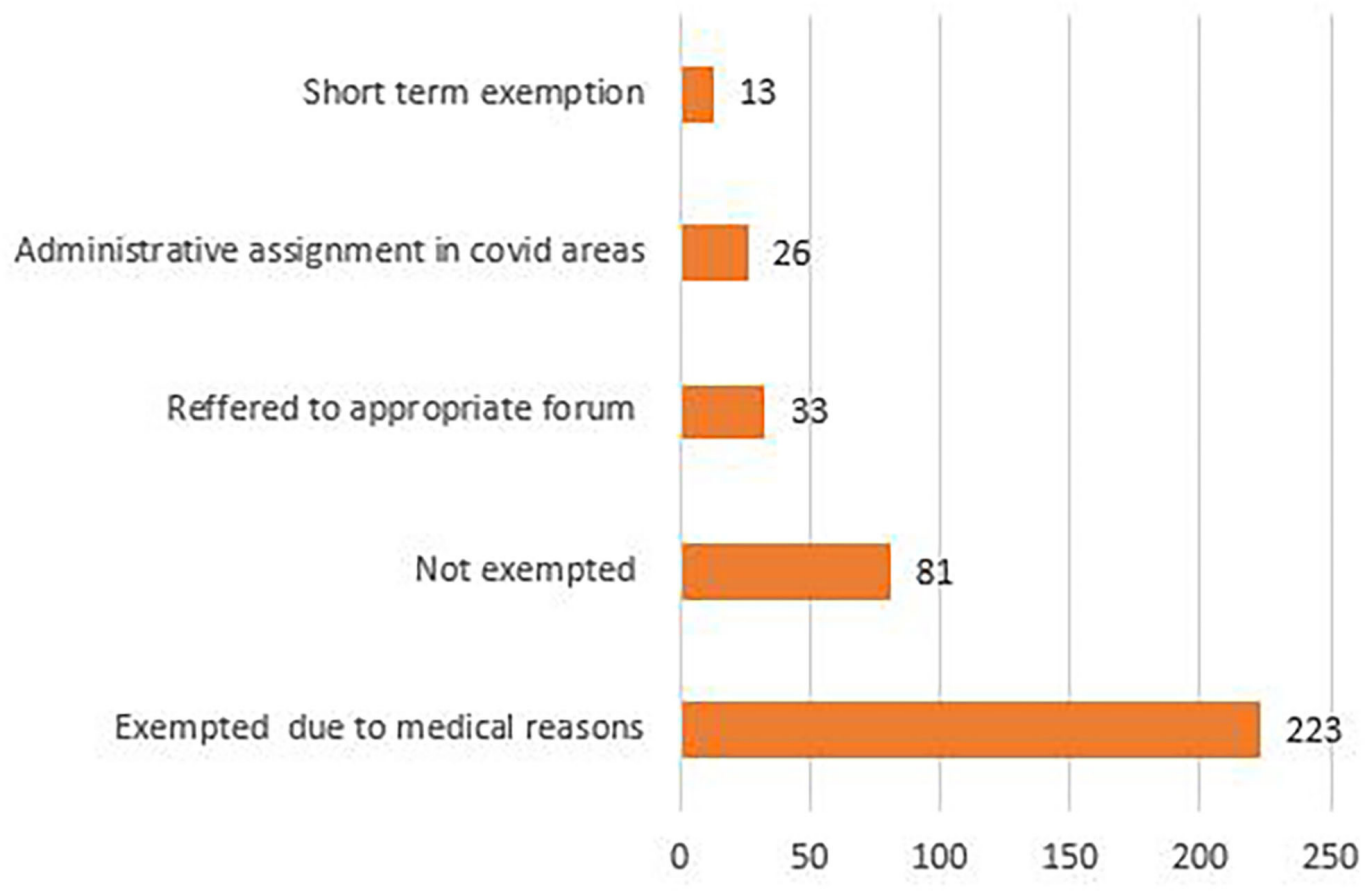
Decision on exemption request under medical grounds.

As can be seen in Figure 4, out of the 376 applications, 223 nursing officers were exempted by the medical board and 13 were given a short-term exemption. Twenty six applicants stated reasons not amounting to serious medical or health issues but were exempted from core ward assignment, which included 6 h of continuous duty wearing PPE in the isolation ward, and were rather deployed for administrative duties in COVID areas. Eighty one applicants were not exempted and out of them, 32 were advised to get their medical issues re-evaluated from the respective departments of the institute. Thirty-three applicants were referred to the committee for the management and deployment of nursing staff as their reasons for seeking exemption did not fall under the purview of the medical board.

## Discussion

The institute took all measures effectively in a timely manner to ensure the health and safety of its healthcare providers including nursing staff. Records displayed frequent training and continuous monitoring of infection control measures taken by the nursing officers while performing duties in COVID areas, which reduced the risk of transmitting infection (4). As per the guidelines issued by the Ministry of Health and Family Welfare, GoI, all healthcare personnel who were suffering from chronic illness or having immunocompromised health status or any other high-risk conditions were exempted from duty in COVID care areas.

This study evaluated the reasons stated for exemption by the nursing staff. The medical board constituted by the institute examined the requests, followed the standard national as well as international guidelines, and exempted all nursing officers who had a potential risk of infection due to certain medical conditions from direct COVID patient care. Studies mention that many healthcare workers have conditions that elevate the risk for severe infection or death if they become infected with COVID-19, so organizations will need to decide whether such workers, including physicians, should be redeployed away from highest risk sites. It is not possible to entirely eliminate risks, but prudent adjustments may be warranted (5). Another study states that at the same time, evidence is mounting that preexisting conditions such as cardiovascular disease, diabetes, hypertension, and obesity, as well as older age, are associated with higher risk of hospitalization, admission to an intensive care unit, and death among those with COVID-19 (6, 7).

The study also revealed that highest number of requests for exemption was received from nursing officers in the age group of 25–34 years. This may be attributed to reproductive age group period, as 40 staff members in this group requested exemption either because of pregnancy or they were in the lactation phase, and a good number of them were exempted. Another reason could be anxiety associated with deployment in infectious disease areas (COVID wards) in this age group as compared to the more experienced staff above 34 years of age, and the same is mentioned in other studies (8, 9). In order to overcome the anxiety aspect, multidisciplinary support can be provided through psychological counselors *via* hot line teams & virtual clinics. Adequate availability of PPE must be ensured. Places to relax post duty, basic hygiene needs and nutritious food must also be arranged (10, 11). Most of the requests were received from staff with medical issues related to dual specialities. Sixty percent of the total applicants were exempted, while the others were given temporary exemption especially in the case of lactating mothers. The exempted staff members were deployed in non-COVID patient care areas so optimal utilization of resources could be done. The personal issues consisted mainly of ailing family member with no support system and fear of passing on the infection as the nursing staff members were also the primary care giver at their homes. Although counseling was done to some extent in these cases, the requests were redirected to senior nursing management to provide support and arrange for counseling to resolve the personal issues.

It was difficult to relate these findings to other institutions/hospitals as the literature on this topic is scarce.

## Conclusion

In these unprecedented times, frontline workers are the army deployed the world over to tackle the enemy, but their protection and safety are paramount while treating patients. Numerous mechanisms were developed to ensure the protection of staff members both in terms of physical and mental health. Equally important is ensuring the safety of immunocompromised staff and their engagement in non-COVID areas to ensure optimal and rational staff deployment in both COVID and non-COVID patient care settings. The overall health of healthcare providers is a vital chain link for effective and efficient management of pandemics, and there have to be clear guidelines for vulnerable nursing staff regarding their deployment in direct patient care.

## Data availability statement

The raw data supporting the conclusions of this article will be made available by the authors, without undue reservation.

## Ethics statement

Written informed consent was obtained from the individual(s) for the publication of any potentially identifiable images or data included in this article.

## Author contributions

SS, ND, AK, and SB contributed to conceptualization and design of the study. SKS contributed in data analysis. SS wrote the first draft of the study. ND, AK, NP, and SB revised and approved the manuscript. All authors contributed to the article and approved the submitted version.

## Conflict of interest

The authors declare that the research was conducted in the absence of any commercial or financial relationships that could be construed as a potential conflict of interest.

## Publisher's note

All claims expressed in this article are solely those of the authors and do not necessarily represent those of their affiliated organizations, or those of the publisher, the editors and the reviewers. Any product that may be evaluated in this article, or claim that may be made by its manufacturer, is not guaranteed or endorsed by the publisher.
