# Supplementary material for: Nursing personnel management during COVID-19 pandemic: An exemption trend in view of health reasons
Source: Front Public Health. 2022 Oct 28;10:961308. doi: 10.3389/fpubh.2022.961308 (PMC9650431; doi:10.3389/fpubh.2022.961308)
Supplement: Supplementary file 1 [file Data_Sheet_1.docx]

**Table 1**. Nursing staff allocation for 250 Bed COVID 19 treatment facility.

| Designation of Nursing staff | Number |
| --- | --- |
| Nursing Superintendent | 1 |
| Deputy Nursing Superintendent | 1 |
| Assistant Nursing Superintendent | 8 |
| Senior Nursing officers | 20 |
| Nursing Officers | 220 |

**Table 2**. Area wise distribution of nursing manpower in COVID 19 treatment facility

| Type of Patient Care Area | No of Beds | Nursing staff deployed for 24 hour shift duty (4 shifts of 6 hours each) |
| --- | --- | --- |
| Intensive Care Unit (ICU) | 50 | 100 |
| High Dependency Unit (HDU) | 120 | 96 |
| Single Bed Isolation wards | 80 | 40 |
